# High-volume infiltration analgesia in bilateral hip arthroplasty: A randomized, double-blind placebo-controlled trial

**DOI:** 10.3109/17453674.2011.596063

**Published:** 2011-09-02

**Authors:** Lasse Ø Andersen, Kristian S Otte, Henrik Husted, Lissi Gaarn-Larsen, Billy Kristensen, Henrik Kehlet

**Affiliations:** ^1^Departments of Anesthesiology; ^2^Orthopedic Surgery, Hvidovre Hospital; ^3^Section of Surgical Pathophysiology, Rigshospitalet, Copenhagen University; ^4^The Lundbeck Center for Fast-track Hip and Knee Arthroplasty, Hvidovre Hospital, Copenhagen, Denmark; Correspondence: lasseandersen@email.dk

## Abstract

**Background and purpose:**

High-volume infiltration analgesia may be effective in postoperative pain management after hip arthroplasty but methodological problems prevent exact interpretation of previous studies.

**Methods:**

In a randomized, double-blind placebo-controlled trial in 12 patients undergoing bilateral total hip arthroplasty (THA) in a fast-track setting, saline or high-volume (170 mL) ropivacaine (0.2%) with epinephrine (1:100,000) was administered to the wound intraoperatively along with supplementary postoperative injections via an intraarticular epidural catheter. Oral analgesia was instituted preoperatively with a multimodal regimen (gabapentin, celecoxib, and acetaminophen). Pain was assessed repeatedly for 48 hours postoperatively, at rest and with 45° hip flexion.

**Results:**

Pain scores were low and similar between ropivacaine and saline administration. Median hospital stay was 4 (range 2–7) days.

**Interpretation:**

Intraoperative high-volume infiltration with 0.2% ropivacaine with repeated intraarticular injections postoperatively may not give a clinically relevant analgesic effect in THA when combined with a multimodal oral analgesic regimen with gabapentin, celecoxib, and acetaminophen.

Continuous epidural analgesia (Choi et al. 2003) or continuous or single-shot peripheral nerve blocks (Boezaart 2006, Ilfeld et al. 2008) may provide sufficient analgesia after total hip arthroplasty (THA), but both techniques are associated with potential motor blockade, thereby hindering early rehabilitation (Choi et al 2003, Boezaart 2006, Ilfeld et al. 2008).

Local infiltration analgesia (LIA) (Röstlund and Kehlet 2007, Kerr and Kohan 2008, Otte et al. 2008) with intraoperative infiltration of local anesthetic in the surgical wound and subsequent supplementary postoperative intraarticular or wound injections has been reported to be effective in knee arthroplasty (Andersen et al. 2008). However, for THA only limited and inconclusive data are available from placebo-controlled and randomized trials (Bianconi et al. 2003, Andersen et al. 2007 a, b, Busch et al. 2010) and from non-randomized cohort studies (Kerr and Kohan 2008, Otte et al. 2008). We therefore decided to evaluate the analgesic efficacy of LIA in a placebo-controlled, randomized and double-blind trial in fast-track bilateral hip arthroplasty with administration of either ropivacaine or saline to the wound, thereby limiting the large inter-individual pain response to THA. This design has proven valid in assessing the analgesic value of LIA in TKA (Andersen et al. 2008). The primary endpoint was pain on flexion of the hip joint 8 hours postoperatively.

## Patients and methods

The study was approved by the local ethics committee (Copenhagen and Frederiksberg, Denmark, Reg.no.KF 01 2006-4062) and the Danish Data Protection Agency (Copenhagen, Denmark) and was registered with ClinicalTrials.gov under the US National Library of Medicine (Code NCT 00864409). The study was performed in compliance with the Helsinki Declaration.

12 consecutive patients scheduled for total bilateral hip arthroplasty (THA) were included from October 2006 through April 2009. The patient characteristics were: 7 men/5 women, mean age 60 (range 41–82) years, mean weight 78 (63–106) kg, mean BMI 26 (23–31), American Society of Anesthesiologists physical status I/II = 7/5. All patients received intraoperative periarticular infiltration with 170 mL 0.2% ropivacaine and epinephrine (1:100,000) in one hip (Otte et al. 2008), and similar infiltration with 170 mL 0.9% saline in the opposite hip. Patients who were scheduled for bilateral THA and who were able to understand and speak Danish and able to give informed oral and written consent were included in the study. Exclusion criteria were treatment with opioids or steroids, rheumatoid arthritis or other immunological diseases, history of stroke or any neurological or psychiatric disease potentially influencing pain perception (e.g. depression, diabetic neuropathy etc.), allergies to any of the drugs administered, and BMI > 40. All inclusions and data registrations were done by one of two investigators, anesthetic procedures were performed by one of two anesthesiologists, and all patients were operated by one of two surgeons. A computer-generated random sequence concealed in opaque sealed envelopes, which were consecutively opened on the morning of surgery, determined which side would receive active treatment. The medicine used for each individual patient was prepared by an investigator not otherwise involved in patient data collection.

### Procedures

Surgery was performed under spinal anesthesia with 15 mg plain bupivacaine (3 mL, 5 mg/mL) and propofol was administered (0.5–5 mg/kg/h) for sedation if required. Low-molecular-weight heparin (4,500 U) was administered subcutaneously 6–8 hours postoperatively for thromboprophylaxis and once daily until discharge. A standardized intraoperative regimen for fluid administration was used with 0.9% saline (5 mL/kg/h) and colloid (Voluven; 7.5 ml/kg/h) (Holte et al. 2007). Bilateral THA was performed sequentially through similar standard posterior approaches without the use of minimally invasive surgical techniques. Drains were not used.

In one hip, the patients received infiltration with 340 mg ropivacaine (0.2%) with adrenaline (1:100.000) in a total volume of 170 mL. The mixture was injected using a systematic technique to ensure uniform delivery of the local anesthetic to all the tissue that was incised or instrumented during the procedure (Otte et al. 2008). The first 50 mL was injected in the periacetabular tissues after reaming of the acetabulum and before insertion of the acetabular component. After insertion of the femoral component, another 50 mL was evenly injected into the cut rotators and the gluteus muscles and the lateral part of the femoral muscle where the guide-pin had been placed. An epidural catheter (18G) with 3 holes was inserted 10 cm distal to the incision and tunneled into the hip joint with placement of the tip near the femoral head, after which the capsule was closed and 20 mL was injected into the catheter to ensure that there was no kinking. Finally, 50 mL was injected fanwise into the subcutaneous layers. To minimize the risk of cutaneous blister formation, the subcutaneous injections did not contain epinephrine. The opposite hip received identical infiltrations with 170 mL 0.9% saline and similar placement of a catheter.

Postoperatively, the patients were transferred to the post-anesthesia care unit (PACU) and then to a specialized knee and hip arthroplasty unit with a well-defined multimodal fast-track rehabilitation regimen (Husted et al. 2008).

All patients received orally administered celecoxib (200 mg every 12 hours; 400 mg preoperatively), slow-release paracetamol (2 g every 12 hours), gabapentin (600 mg at 0800 h and 300 mg at 1600 h daily, initiated preoperatively along with patient-controlled analgesia (PCA) for 48 h with intravenous morphine (20 µg/kg), lock-out set to 10 min. Apart from administration of additional sufentanil or morphine in the PACU, the morphine PCA was the only opioid administered in the 48-h study period.

8 hours postoperatively, a 20-mL injection of the drug mixture (40 mg ropivacaine and epinephrine (1:100.000)) or 0.9% saline was administered intraarticularly through the catheters in accordance with randomization. 24 h postoperatively, 50 mL of the drug mixture (100 mg ropivacaine with epinephrine (1:100.000)) or 50 mL 0.9% saline was injected.

### Data collection

The primary endpoint was postoperative pain in each hip 8 h postoperatively, which was assessed using a visual analog scale (VAS) from 0 to 10 cm, with 0 indicating no pain and 10 indicating the worst possible pain, at rest and upon 45° flexion of the hip with the leg straight. Pain assessment was performed by one of two investigators who were blinded regarding the randomization.

Pain was recorded at 4, 8, 9, 24, 24.5, 25, 26, 32, and 48 h after surgery. Throughout the 48-h study period, the amount of morphine delivered via the PCA pump was registered. Length of hospital stay (LOS) was also registered. All the patients were discharged directly to their homes according to functional discharge criteria: ability to get in and out of bed, to get dressed, and to get into and up from a chair; ability to walk independently for 50 m with appropriate walking aids; and acceptance of discharge.

### Statistics

The number of participants was set to 12, since no meaningful power calculation could be performed from published data in unilateral hip arthroplasty. Tests for significant differences between treatment groups were done using the Wilcoxon signed ranks test. All p-values < 0.05 were considered statistically significant. All data analysis was done using SPSS for Windows version 12.0.

## Results

VAS pain scores were similar for the hip infiltrated with ropivacaine and epinephrine and the hip infiltrated with saline (p > 0.05), both at rest and with hip flexion, except for significantly less pain at rest 32 h postoperatively in the hip infiltrated with saline (p = 0.03) (Figure).

**Figure:**
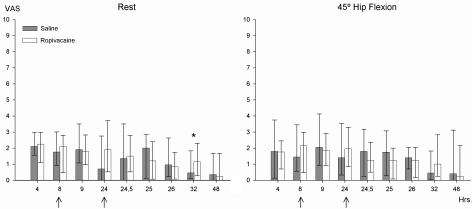
Postoperative pain after bilateral hip arthroplasty (n = 12) with local infiltration of ropivacaine or saline intraoperatively. Re-injection of ropivacaine or saline after 8 h (20 mL/40 mg) and 24 h (50 mL/100 mg). Values are median with twenty-fifth and seventy-fifth percentiles. VAS: visual analog scale, where 0 cm = no pain and 10 cm = worst pain. * p < 0.05. The arrows indicate re-injection times.

In the PACU, intravenous administration of sufentanil was median 10 (interquartile range 0–30) µg, and median cumulative postoperative PCA morphine administration was 6.5 (4–12) mg, 14 (11–26) mg, 32 (17–53) mg, 36 (19–61) mg, and 43 (20–70) mg at 4, 8, 24, 32, and 48 h postoperatively. Mean duration of surgery was 178 (range 76–325) min. Median hospital stay was 4 days (range 2–7).

No clinical side effects, including cardiac and hemodynamic changes requiring intervention, were observed during the study period (intraoperatively and 0–48 h postoperatively).

## Discussion

We found that there was acceptable pain relief with multimodal oral analgesia without any clinically relevant analgesic effect of intraoperative high-volume administration of local anesthetic and adrenaline with supplementary postoperative injections to the wound. In spite of the fact that there have been relatively few positive trials published so far (Reilly et al. 2005, Busch et al. 2006, Vendittoli et al. 2006, Andersen et al. 2007a, b, 2008, Toftdahl et al. 2007) high-volume infiltration analgesia is increasingly used in major joint replacement surgery. Although postoperative administration of local anesthetic to the wound may be effective in many surgical procedures (Liu et al. 2006), our results emphasize the need for procedure-specific placebo-controlled trials.

The choice of postoperative analgesic technique after THA may include continuous or single-dose peripheral nerve blocks or continuous epidural analgesia (Fischer et al 2005, Ilfeld et al. 2008), high-volume local infiltration analgesia (LIA) (Andersen et al. 2007a, b), continuous local administration of anesthetic to the wound (Bianconi et al. 2003), or a multimodal analgesic regimen with COX-2 inhibitor or conventional NSAID, paracetamol, and gabapentin (Tiipana et al. 2007), with weak or strong opioids as rescue analgesics. The choice between these analgesic techniques depends on efficacy, side effects, costs, and demand for expertise. Our study has shown that there is acceptable postoperative pain relief with a multimodal parenteral regimen with COX-2 inhibitor, gabapentin, and paracetamol—with no additional pain relief using the high-volume local infiltration analgesia technique. These findings correspond to those in prospective, large-scale observational series after THA discharge (Andersen et al. 2009). Among the techniques mentioned, a multimodal non-opioid analgesic regimen may be preferable compared to epidural analgesia or peripheral femoral or posterior lumbar plexus block, since these techniques may be associated with motor side effects with potentially delayed postoperative mobilization.

Although previous THA trials have assessed the value of the high-volume infiltration technique compared to placebo (Andersen et al. 2007b) or epidural analgesia (Andersen et al. 2007a), and found reduced pain and opioid requirements along with improvement in physical function 1 week after surgery, these trials did not incorporate the multimodal analgesic regimen used in this trial, although an NSAID was part of the high-volume infiltration mixture. Furthermore, interpretation of some of these trials is hindered by the fact that NSAID was only given in the LIA group but not in the control group (Andersen et al. 2007a,b, Busch et al. 2010), since NSAID per se is effective in THA (Fischer et al 2005). Due to the design of our study, morphine requirements could not be assessed but the overall PCA morphine consumption was minimal (about 40 mg during the first 48 h) despite having bilateral THA. Although the high-volume infiltration analgesia technique apparently does not provide additional analgesia when combined with an effective multimodal analgesic regimen in total hip arthroplasty, further studies evaluating the LIA technique with other oral analgesic regimes may be of interest. Furthermore, the optimal site for administration of local anesthetic has not been determined, although continuous infusion of ropivacaine to the subcutaneous layer of the wound after THA has been reported to have an analgesic and opioid-sparing effect (Bianconi et al. 2003).

Although acceptable postoperative pain relief may be achieved with a simple oral analgesic regimen, future improvements in early and late postoperative recovery and rehabilitation should be studied regarding the effect of reduction in the neuroendocrine and inflammatory response in THA, where an elevated C-reactive protein may be a predictor of convalescence after discharge from hospital (Hall et al. 2001). Further improvement in postoperative analgesia and recovery may therefore be achieved with administration of glucocorticoids, as demonstrated in preliminary trials (Romundstad et al 2004, Kardash et al. 2008).

Since the results reported in this study could possibly be the result of a type-2 error (i.e. a false negative result) due to the small number of observations, larger-scale trials may be needed to confirm our results. However, the inclusion of patients undergoing bilateral THA increases the strength of our data, and a study of similar design found a substantial analgesic effect of LIA in bilateral knee arthroplasty (Andersen et al. 2008). Finally, the low pain scores achieved with the multimodal oral analgesia make it unlikely that a clinically important analgesic effect was overlooked.

In conclusion, the results from this randomized, double-blind placebo-controlled trial in bilateral THA showed that there was no clinically relevant analgesic effect of intraoperative high-volume infiltration analgesia with 0.2% ropivacaine combined with repeated postoperative wound injections. Acceptable postoperative pain relief may be achieved with a multimodal oral analgesic regimen with gabapentin, celecoxib, and paracetamol.
